# Circulating Levels of Soluble α-Klotho and FGF23 in Childhood Cancer Survivors: Lack of Association with Nephro- and Cardiotoxicity—A Preliminary Study

**DOI:** 10.3390/jcm13102968

**Published:** 2024-05-17

**Authors:** Kacper Kozłowski, Katarzyna Konończuk, Katarzyna Muszyńska-Rosłan, Beata Żelazowska-Rutkowska, Katarzyna Taranta-Janusz, Katarzyna Werbel, Maryna Krawczuk-Rybak, Eryk Latoch

**Affiliations:** 1 Department of Pediatric Oncology and Hematology, Medical University of Bialystok, 15-274 Białystok, Poland; kozlowskikacper@gmail.com (K.K.); kononczukk@gmail.com (K.K.); kmroslan@post.pl (K.M.-R.); maryna.krawczuk-rybak@umb.edu.pl (M.K.-R.); 2 Department of Pediatric Laboratory Diagnostics, Medical University of Bialystok, 15-274 Białystok, Poland; zelazowskab@wp.pl; 3 Department of Pediatrics and Nephrology, Medical University of Bialystok, 15-274 Białystok, Poland; katarzyna.taranta@wp.pl; 4 Department of Neonatology, Pathology and Newborn Intensive Care, Jędrzej Śniadecki Independent Public Healthcare Center—Regional Hospital, 15-027 Białystok, Poland; katarzynawerbel@gmail.com

**Keywords:** cardiovascular disease, children, chronic kidney disease, fibroblast growth factor-23, late effects, leukemia, lymphoma, nephrotoxicity, solid tumor

## Abstract

**Background/Objectives:** The survival rate among pediatric cancer patients has reached 80%; however, these childhood cancer survivors (CCSs) are at a heightened risk of developing chronic conditions in adulthood, particularly kidney and cardiovascular diseases. The aims of this study were to assess the serum α-Klotho and FGF23 levels in CCSs and to determine their association with nephro- and cardiotoxicity. **Methods:** This study evaluated a cohort of 66 CCSs who remained in continuous remission, with a mean follow-up of 8.41 ± 3.76 years. **Results:** The results of this study revealed that CCSs exhibited significantly higher levels of soluble α-Klotho compared to healthy peers (1331.4 ± 735.5 pg/mL vs. 566.43 ± 157.7 pg/mL, *p* < 0.0001), while no significant difference was observed in their FGF23 levels. Within the participant cohort, eight individuals (12%) demonstrated a reduced estimated glomerular filtration rate (eGFR) below 90 mL/min/1.73 m^2^. The relationship between treatment with abdominal radiotherapy and reduced eGFR was confirmed (*p* < 0.05). No correlations were found between potential treatment-related risk factors, such as chemotherapy or radiation therapy, serum levels of α-Klotho and FGF23, and nephro- and cardiotoxicity. **Conclusions:** In conclusion, this preliminary cross-sectional study revealed elevated levels of α-Klotho among childhood cancer survivors but did not establish a direct association with anticancer treatment. The significance of elevated α-Klotho protein levels among CCSs warrants further investigation.

## 1. Introduction

In the past few years, the overall survival rate among children treated for cancer has risen to 80% as a result of improved multimodal treatment methods. However, many studies emphasize that childhood cancer survivors (CCSs) are at risk of developing multiple chronic diseases earlier in life. In fact, large population-based studies indicate that over 60% of CCSs suffer from at least one chronic condition many years after completion of therapy [1]. Anticancer treatment used in childhood can cause a wide range of health problems—from minor health issues to multiple-organ or system failure. Consistently, health problems lead to a significant decrease in quality of life and lifespan.

Some of the most common long-term sequelae include genitourinary diseases, with a frequency ranging from 10 to 80%, depending on the cohort studied [2]. Damage to the genitourinary system can result not only from the cancer’s localization but also from the nephrotoxic drugs administered during treatment. Agents with the greatest nephrotoxic potential include cisplatin, carboplatin, ifosfamide, and methotrexate. The mechanism of damage varies depending on the specific drug and can impair the glomeruli, tubules, and urinary bladder mucosa [3]. Additionally, abdomen irradiation significantly reduces renal function and harms the tissues of the urinary system and their functionality. Supportive advanced treatment is also becoming increasingly important in kidney damage. These encompass certain antibiotics like aminoglycosides, amphotericin, or vancomycin, alongside diuretics, analgesics, and contrast agents. Many of the long-term effects can occur at different ages and manifest initially as a decrease in glomerular filtration rate, a loss of tubular function, and the occurrence of hypertension but can lead up to the onset of chronic kidney disease (CKD) and renal failure [4]. 

Commonly used methods to assess renal function have many limitations. The estimated glomerular filtration rate (eGFR) based on serum creatinine (sCr) is now the most important indicator for assessing kidney function. However, its levels are affected by many factors, including lean body mass, the amount of protein in the diet, gender, and hydration status. Despite remarkable medical advances, predicting which CCSs will develop kidney disease remains challenging. The search for markers of early kidney damage to identify patients at risk before the onset of symptoms is therefore a major focus. In recent years, several biomarkers indicating early renal damage have been introduced, especially in the context of developing acute kidney injury (AKI). However, studies on these markers are also increasingly being extended to understand the mechanisms of early kidney injury in the pathogenesis of CKD [5]. Notable among these are soluble α-Klotho and fibroblast growth factor-23 (FGF23), which show promise as biomarkers for chronic kidney disease. They play essential roles in the regulation of bone metabolism, and both are affected following renal failure [6].

Another significant late effect of childhood cancer treatment is cardiovascular disease (CVD), which is highly prevalent and the leading cause of death among cancer survivors. The correlation between CVD and cancer reveals a convergence of common risk factors and pathophysiological mechanisms that potentially increase susceptibility to both conditions [7,8]. Additionally, certain anticancer therapies, such as anthracyclines and radiotherapy, are known to induce cardiovascular toxicity, further increasing the risk of CVD among cancer survivors [9].

Soluble α-Klotho is released as the extracellular domain of membrane α-Klotho, which is highly expressed in kidneys and plays a role in the pathophysiology of many diseases, such as CKD, cancer, and cardiovascular disease [10]. α-Klotho shows its pleiotropism through antioxidant, anti-aging, or anti-apoptotic effects [11]. Fibroblast growth factor-23 is a phosphaturic hormone secreted primarily by bone osteocytes and osteoblasts and regulates the renal reabsorption of phosphate, sodium, and calcium, as well as the expression of α-Klotho [12]. Circulating FGF23 exerts its effects on the kidney by binding to FGF receptors and the α-Klotho receptor to promote phosphaturia and reduce circulating vitamin D levels. Changes in these proteins can directly influence other parameters of mineral metabolism and may also impact kidney function, as well as cause injury to other organs such as the heart, bones, or blood vessels [13]. The combination of elevated FGF23 and Klotho deficiency contributes to cardiovascular diseases in patients with CKD. Fibroblast growth hormone-23 primarily contributes to left ventricle hypertrophy (LVH) and heart failure, while α-Klotho deficiency predominantly promotes arterial calcification and atherosclerosis in conjunction with hyperphosphatemia [14].

The existing literature highlights a gap in understanding the involvement of FGF23 and α-Klotho in pediatric cancer patients. Studies on adults suggest that α-Klotho exhibits inhibitory effects on several cancers, including breast, colorectal, lung, prostate, and hepatocellular carcinoma, and other malignancies. Moreover, elevated α-Klotho expression or activity often correlates with a more favorable prognosis. Additionally, FGF23 and phosphate have emerged as relevant factors in cancer pathophysiology, particularly in cancer types with implications for bone health such as bone sarcoma, multiple myeloma, or those associated with bone metastases. However, the relationship between FGF23 and α-Klotho levels and treatment outcomes remains unclear. Limited evidence suggests that oncogenic osteomalacia in breast cancer may be attributed to molecular alterations in the FGF/FGFR signaling pathway, leading to the overexpression of FGF23 [15,16].

The aim of this study was to investigate the impact of childhood cancer treatment on the levels of α-Klotho and FGF23 and to assess their potential associations with renal and cardiovascular function in children several years after anticancer treatment. 

## 2. Materials and Methods

This study included 66 childhood cancer survivors in continuous remission aged 14.22 ± 4.48 years (29 males and 37 females) treated previously at the Department of Pediatric Oncology and Hematology of Medical University of Bialystok. The study group consisted of children treated for leukemia, lymphoma, and solid tumors (neuroblastoma, nephroblastoma, hepatoblastoma, and germ tumors). The inclusion criteria comprised individuals under 18 years of age with diagnosed cancer, in complete remission, and possessing comprehensive clinical data. The exclusion criteria included bone metastases at diagnosis, cancer recurrence, inflammation, congenital kidney and/or bone disorders, and lack of consent for study participation.

The mean time from the end of treatment was 8.41 ± 3.76 years. The characteristics of the patients are shown in Table 1. The data on age, sex, type of diagnosis, and treatment were collected from medical records. All participants underwent a clinical examination, and anthropometric characteristics were collected during follow-up visits using standard techniques. Body mass index (BMI) was calculated by dividing weight (kg) by height^2^ (m^2^). Blood pressure (BP) (systolic and diastolic) was measured three times using a standardized sphygmomanometer, performed three times at 1–2-min intervals; the participant rested peacefully for 5 min before the measurement. Hypertension was defined as a mean systolic BP and/or diastolic BP level ≥ 95th percentile adjusted for age, sex, and height. Left ventricular ejection fraction (EF) and shortening fraction (SF) were assessed by a pediatric cardiologist using echocardiography. The survivors were not taking vitamin D or phosphorus binders during the study. The control group included 20 healthy peers, children of hospital employees, with no history of cancer and kidney disease.

Fasting peripheral blood was collected after a 12 h interval for routine laboratory tests. The samples were stored at −80 °C for further analysis. The serum soluble Klotho and plasma C-terminal FGF23 levels were measured using an enzyme-linked immunosorbent assay (ELISA) kit (IBL, International GmbH, Hamburg, Germany; intra-assay). Basic laboratory tests were performed using standard methods. Estimated GFR was calculated using the updated Schwartz formula: eGFR = 0.413 × (height in cm/serum creatinine in mg/dL). The stages of chronic kidney disease (CKD) were classified according to the Kidney Disease: Improving Global Outcomes (KDIGO) guidelines as structural or functional abnormalities in renal function for at least 3 months, with or without decreased eGFR or eGFR below 60 mL/min/1.73 m^2^ for at least 3 months, with or without kidney injury.

A statistical analysis was performed using Statistica 13.3. The Shapiro–Wilk test was used to examine normal distribution. The data were expressed as means ± standard deviation (SD), or median and quartiles when appropriate. The Mann–Whitney U test was applied to compare independent variables without normal distribution. The Χ^2^ test was used to compare numerical variables. A correlation analysis between parameters was assessed using Spearman’s rank correlation coefficient. The level of statistical significance was defined as *p* < 0.05. 

## 3. Results

The clinical characteristics of the study group are presented in Table 1. The mean age at the time of the study was 14.22 ± 4.48 years, and the mean follow-up time from the cessation of the treatment was 8.41 ± 3.76 years. All subjects presented normal renal functions at the time of diagnosis.

**Figure 1:**
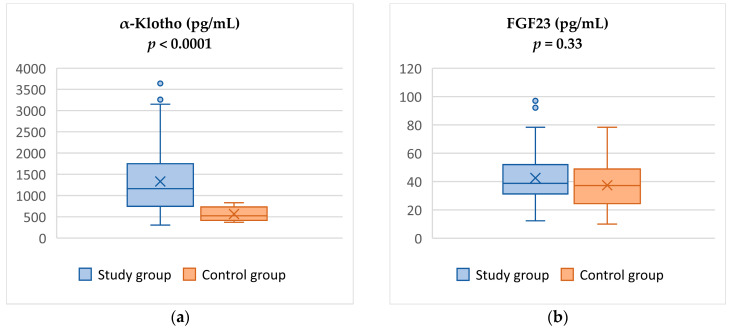
Comparison of α-Klotho (**a**) and FGF23 levels (**b**) between study and control groups.

The study group presented significantly higher α-Klotho levels (1331.4 ± 735.5 pg/mL vs. 566.43 ± 157.7 pg/mL, *p* < 0.0001) than the control group. In contrast, we did not observe any differences in FGF23 concentration between the analyzed groups (42.6 ± 18.9 pg/mL vs. 37.4 ± 16.7 pg/mL, *p* = 0.334)—Figure 1.

We observed no difference in α-Klotho levels according to sex in both the study (female: 1469.8 ± 821.73 pg/mL vs. male: 1154.83 ± 574.4 pg/mL, *p* = 0.15) and control (female: 554.12 ± 168.6 pg/mL vs. male: 588.73 ± 151.56 pg/mL, *p* = 0.57) groups. Similarly, no differences were found in the FGF23 levels between sexes in the study group (female: 42.02 ± 17 pg/mL vs. male: 43.2 ± 21.4 pg/mL, *p* = 0.9) or control group (female: 38.8 ± 20.6 pg/mL vs. male: 36 ± 12.8 pg/mL, *p* = 0.72).

We divided the study participants into two groups based on their eGFR. The first group comprised 8 survivors with eGFR below 90 mL/min/1.73 m^2^, while the second group included 58 participants with eGFR above 90 mL/min/1.73 m^2^. Except for serum creatinine (0.86 ± 0.09 mg/dL vs. 0.57 ± 0.17 mg/dL, *p* < 0.001), the biomarkers studied did not differ between groups: α-Klotho (1251.3 ± 714.90 pg/mL vs. 1615.75 ± 959.70 pg/mL, *p* = 0.21), FGF23 (43.50 ± 12.8 pg/mL vs. 43.9 ± 21 pg/mL, *p* = 0.93), and urinary creatinine (uCr) (138.92 ± 70.00 mg/dL vs. 100.38 ± 51.12 mg/dL, *p* = 0.12). The results are shown in Table 2.

Participants were also divided based on their initial diagnosis and treatment as follows: leukemia (n = 46), Hodgkin lymphoma and non-Hodgkin lymphoma (n = 5), and solid tumors (n = 15). No significant differences in levels of α-Klotho (*p* = 0.19), FGF23 (*p* = 0.75), sCr (*p* = 0.17), and uCr (*p* = 0.55) were found between the subgroups.

The study group was further stratified based on the time of treatment cessation. The 20 individuals who had ended treatment over 10 years prior had significantly lower sCr levels (0.74 ± 0.15 mg/dL vs. 1.48 ± 6.18 mg/dL; *p* < 0.001) and significantly higher uCr levels (172.45 ± 75.41 mg/dL vs. 116 ± 57.45 mg/dL, *p* = 0.007) than subjects who had ended treatment less than 10 years prior. In contrast, the levels of α-Klotho protein (1239.5 ± 876.5 pg/mL vs. 1371.4 ± 672 pg/mL, *p* = 0.2) and FGF23 (47.3 ± 17.11 pg/mL vs. 40.5 ± 19.4 pg/mL, *p* = 0.055) did not differ significantly between the groups.

There were no differences between the study participants who received radiation therapy (n = 21) and those who were not irradiated (n = 45) in their α-Klotho (*p* = 0.31), FGF23 (*p* = 0.55), eGFR (0.94), sCr (*p* = 0.21), and uCr levels (*p* = 0.28). The effect of abdomen radiotherapy on the levels of the examined markers in the survivor group was subsequently analyzed. From the examined group, eight patients (12%) received radiotherapy to the abdominal area. They exhibited significantly lower eGFR, albeit within the normal range (100.75 ± 21.86 mL/min/1.73 m^2^ vs. 124.9 ± 28.6 mL/min/1.73 m^2^, *p* = 0.026), while no significant differences were observed in their α-Klotho (*p* = 0.054), FGF23 (*p* = 0.49), sCr (*p* = 0.09), and uCr levels (*p* = 0.96).

Furthermore, the correlations between the cumulative dose of selected drugs and both Klotho and FGF23 were examined. The results are shown in Table 3 and Table 4. The analyses revealed negative correlations between α-Klotho protein and sCr (r = −0.32, *p* = 0.01) and between FGF23 and cumulative methotrexate dose (r = −0.46, *p* = 0.002). We also noted a positive correlation between FGF23 and uCr levels (r = 0.41, *p* < 0.001). Due to the limited number of variables showing a significant effect on Klotho and FGF23 levels in the univariate analysis, it was not possible to develop a reliable analytical model for multivariate analysis. The levels of α-Klotho and FGF23 showed no significant correlation in either the study or control groups.

Subsequently, we conducted a separate analysis specifically for survivors who had undergone unilateral nephrectomy (n = 7). There were no statistically significant differences in α-Klotho and FGF23 levels between childhood cancer survivors (CCSs) with and without simple nephrectomy. Among the 7 patients who underwent nephrectomy, the mean serum α-Klotho protein levels were 1772.26 ± 834.03 pg/mL, compared to 1279.10 ± 712.75 pg/mL in the 59 patients without nephrectomy (*p* = 0.1). Similarly, there was no significant difference in FGF23 levels between these groups (36.64 ± 14.44 pg/mL and 43.30 ± 19.30 pg/mL, respectively, *p* = 0.61).

Further, we compared individuals in terms of α-Klotho and FGF23 protein levels between those who underwent HSCT (n = 10) and those who were not treated with HSCT (n = 56). There were no significant differences between groups (α-Klotho levels: 1413.27 ± 923.27 pg/mL vs. 1312.35 ± 711.70 pg/mL, *p* = 0.92; FGF23: 42.35 ± 17.21 pg/mL vs. 42.42 ± 19.40 pg/mL, *p* = 0.81).

Finally, considering the extensive body of research highlighting the link between α-Klotho and FGF23 with the cardiovascular system, we explored the relationship of these proteins with blood pressure, left ventricular ejection fraction (EF), and shortening fraction (SF). The data obtained revealed no significant correlation between α-Klotho and the parameters assessed by echocardiography (for SF: r = −0.036, *p* = 0.81; for EF: r = −0.009, *p* = 0.95). Similarly, we observed no correlation between FGF23 and echocardiography measurements (for SF: r = −0.20, *p* = 0.18; for EF: r = −0.24, *p* = 0.1).

## 4. Discussion

The etiology of late nephrotoxicity in childhood cancer survivors remains elusive. While advances have been made in understanding treatment-related factors implicated in acute kidney injury, certain factors also predispose individuals to chronic kidney disease in subsequent years. Notably, a subset of childhood cancer survivors, despite having no history of acute kidney injury, may nonetheless develop chronic kidney disease long after the completion of treatment. Research indicates that individuals who have undergone treatment are nine times more likely to develop kidney failure than their siblings [3]. In recent times, endeavors have been undertaken to discern cancer survivors who exhibit susceptibility to kidney failure several years after cancer treatment. Currently, new markers of kidney damage are being sought that can herald kidney damage even before symptoms appear [17,18]. 

The objective of this study was to investigate whether childhood cancer treatment affects the levels of soluble α-Klotho and fibroblast growth factor-23 in survivors of childhood cancer several years after treatment, as well as their relationship to kidney damage and cardiovascular disease. In the present study, a significant increase in α-Klotho levels compared to the reference group were found. However, besides a negative correlation observed between α-Klotho and serum creatinine levels, no correlation was found between estimated glomerular filtration rate and the cytostatics used. Reviewing the literature, no data were found on α-Klotho protein in children with cancer or in survivors.

The experiments initially conducted on mice with the hypomorphic α-Klotho gene demonstrated the anti-aging properties of the α-Klotho protein. These mice exhibited a range of detrimental effects, including a shortened lifespan, impaired growth, renal disease, cardiovascular and respiratory disease, cognitive impairment, and fibrosis. Conversely, the overexpression of α-Klotho resulted in opposite effects—prolonging lifespan. In humans, α-Klotho levels decline with age and are associated with, among other things, chronic kidney disease and many of the most common diseases characteristic of the aging population in general, such as diabetes, cardiovascular complications, etc. [19].

The role of α-Klotho in the pathogenesis of chronic kidney disease, however, is debatable [20]. Klotho exhibits strong expression in the kidney, with its level being closely associated with renal function, as indicated by studies [21]. Consequently, Klotho deficiency is suggested to be a prevalent characteristic of kidney disorders [22], playing a significant role in their etiology and progression, including CKD and its associated complications. A decline in soluble α-Klotho levels has been noted in the initial stages of CKD, preceding the rise in serum creatinine levels, and this reduction progresses with the advancement of CKD [23,24]. Notably, diminished α-Klotho levels have been linked to heightened risks of adverse clinical outcomes in CKD patients, such CKD progression, mortality, and mineral bone disorders [25,26]. 

Evidence from the literature suggests that a deficiency in soluble α-Klotho contributes to arterial calcification and atherosclerotic disease, either independently or through concomitant hyperphosphatemia. Moreover, reduced concentrations may attenuate the adverse cardiovascular effects of FGF23 [14]. In our study, we conducted an analysis to explore the relationship between α-Klotho protein levels and hypertension, as well as ejection fraction measured by echocardiography. However, our findings did not reveal any significant association between α-Klotho protein levels and these cardiovascular parameters. One possible explanation for this lack of association could be the relatively young age of the childhood cancer survivors included in our study. It is plausible that the effects of α-Klotho protein on hypertension and ejection fraction may manifest differently in younger individuals compared to older adults. Further research involving a broader age range of participants may provide deeper insights into the complex interplay between α-Klotho protein levels and cardiovascular health outcomes in childhood cancer survivors.

The elevated levels of α-Klotho protein observed in our study did not support our research hypothesis of the presence of lower levels of the protein in childhood cancer survivors compared to healthy peers. The role of Klotho protein in this specific population remains unknown and requires validation through prospective studies involving large cohorts of patients. Additionally, we find no evidence of an association between α-Klotho protein and nephro- and cardiotoxicity or treatment in childhood cancer survivors.

A large and growing body of literature demonstrates that chronic kidney disease promotes elevated levels of fibroblast growth factor-23 and α-Klotho deficiency, thereby increasing susceptibility to cardiovascular disease [27,28,29]. In this cross-sectional study, we did not confirm an increase in FGF23 levels in the study population and its association with reduced eGFR, hypertension, or abnormal ejection fraction in echocardiography. This is likely attributed to the limited number of patients with chronic kidney disease and cardiovascular abnormalities. However, the purpose of the study was, among other things, to verify whether abnormal FGF23 secretion, which could be indicative of early kidney damage, is already observed at a young age. Moreover, the performed analyses revealed negative correlations between FGF23 and cumulative methotrexate dose, as well as a positive correlation between FGF23 and urine creatinine levels. Yet, the correlation exhibited a weak strength, casting doubt on the reliability of any inferences drawn from it.

A comprehensive study involving 1122 individuals aged 18 and above who underwent treatment for childhood cancer demonstrated significant long-term renal function outcomes [30]. Findings from the extended evaluation, spanning 35 years, demonstrated that 6.6% of CCSs exhibited reduced eGFR, indicating a heightened risk of renal dysfunction. Moreover, renal function deteriorated progressively over time among CCSs who received nephrotoxic therapy. In our investigation, 12% of participants exhibited decreased eGFR levels. Notably, a correlation between abnormal eGFR and the specific treatment administered was found. This discrepancy may stem from the underrepresentation of individuals with diminished eGFR levels, as well as the relatively short maximum 11-year follow-up period post-treatment.

There are several limitations of this study. It was a single-center analysis with a relatively small number of patients, which may influence the occurrence of bias. The diversity observed in the study group regarding different types of cancer made it challenging to analyze the effects of some specific treatment protocols, especially those associated with rare cancers. Therefore, we focused on evaluating the impact of specific treatments, such as cytostatics and radiation therapy. The predominance of non-solid tumors among patients could potentially influence the study outcomes and warrants consideration. The assessment of cardiotoxicity is also limited by the lack of evaluation of markers of myocardial damage such as N-terminal natriuretic propeptide type B (NT-proBNP) or high-sensitivity troponin T (hs-TnT). Another limitation is that eGFR was estimated from creatinine values, rather than measured with techniques such as plasma clearance of iohexol.

The available literature lacks data on the role of α-Klotho and FGF23 in children treated for cancer. The limited number of published studies conducted to date in adults suggests a significant role of α-Klotho as a tumor suppressor, partially through the inhibition of the IGF-1 and Wnt/β-catenin signaling pathways. Consequently, elevated α-Klotho expression or activity is frequently linked to a more favorable prognosis in various malignancies. Furthermore, FGF23 and phosphate have emerged as pertinent factors in cancer pathophysiology, with FGF23 being particularly relevant for cancer types that predominantly affect bone health (e.g., multiple myeloma) or exhibit bone metastases [15].

In conclusion, to the best of our knowledge, this is the first study to investigate the levels of α-Klotho and FGF23 and their association with nephrotoxicity and cardiotoxicity among childhood cancer survivors several years after treatment. The findings of this pilot cross-sectional study in cancer survivors revealed elevated serum levels of α-Klotho protein among CCSs. However, we did not observe differences in the FGF23 concentrations, nor did we find an association of the investigated markers with nephrotoxicity and cardiovascular dysfunction. The conclusions drawn from this study should be approached with caution, as α-Klotho levels can be influenced by many factors. Further prospective studies involving larger CCS cohorts and longer follow-up periods are needed to collect additional data on the role of the α-Klotho protein in the pathogenesis of renal and cardiovascular diseases, as well as its relationship to cancer.

## Figures and Tables

**Table 1:** Descriptive characteristics of the study group.

	Number (%) ^a^	Mean ± SD ^b^
**Patients**	66 (100)	
Male	29 (43.9)	
Female	37 (56.1)	
Age at diagnosis (years)		5.06 ± 3.60
Age at study (years)		14.22 ± 4.48
Follow-up after treatment (years)		8.41 ± 3.76
Patients with hypertension	18 (27.27)	
Patients without hypertension	48 (72.72)	
**Diagnosis**		
Leukemia	46 (69.7)	
Lymphoma	5 (7.6)	
Solid tumor	15 (22.7)	
**Chemotherapy**		
Methotrexate—cumulative dose (mg/m^2^)	44 (66.7)	11,023.81 ± 7445.70
Cumulative corticosteroid dose (mg/m^2^) ^c^	50 (75.8)	3378 ± 1293.89
Prednisone—cumulative dose (mg/m^2^)	50 (75.8)	1606.22 ± 287.56
Dexamethasone—cumulative dose (mg/m^2^)	42 (63.6)	283.33 ± 163.43
Cyclophosphamide—cumulative dose (mg/m^2^)	48 (72.7)	3702.17 ± 1990.48
Cisplatin—cumulative dose (mg/m^2^)	7 (10.6)	360 ± 132.67
Ifosfamide—cumulative dose (mg/m^2^)	3 (4.5)	5033.33 ± 3000.56
**Radiotherapy**	21 (31.8)	
Cranial radiotherapy (CRT)—cumulative dose (Gy)	11 (16.7)	13.09 ± 2.43
Total body irradiation (TBI)	5 (7.6)	12 ± 0.00
Abdominal radiotherapy (ART)	8 (12.1)	19.95 ± 2.07
Chest radiotherapy (ChRT)	2 (3.0)	19.80 ± 0.00
No	45 (68.2)	
**Nephrectomy**	7 (10.6)	
**HSCT**	10 (15.2)	

^a^ Percent of the total, ^b^ standard deviation (SD), ^c^ calculated as prednisone equivalents. Gy, the Gray; HSCT, hematopoietic stem cell transplantation.

**Table 2:** Comparison of parameters and biochemical factors in childhood cancer survivors according to estimated glomerular filtration ratio (eGFR).

Total	eGFR >90 mL/min/1.73 m^2^	eGFR <90 mL/min/1.73 m^2^	*p* Value
Number of patients	53	8	
Gender (M/F)	21/32	4/4	
Age at start of treatment (years)	5.03 (2.40; 7.02)	4.90 (3.08; 6.41)	0.924
Age at the time of the study (years)	14.2 (10.68; 17.78)	15.31 (14.72; 16.29)	0.487
Follow-up time (years)	8.41 (5.74; 10.27)	9.5 (7.61; 12.08)	0.399
Klotho (pg/mL)	1251 (693.7; 1656.0)	1615 (967.50; 1953.00)	0.205
FGF23 (pg/mL)	43.5 (31.25; 52.41)	43.70 (35.50; 49.65)	0.979
Vitamin D (ng/mL)	19.66 (15.76; 24.1)	22.75 (12.30; 29.93)	0.609
ACR (mg/g)	381.49 (24.07; 131.12)	153.55 (32.04; 239.06)	0.648
Serum creatinine (mg/dL)	0.57 (0.43; 0.66)	0.86 (0.78; 0.96)	0.0001
Urine creatinine (mg/dL)	138.92 (76.81; 187.38)	100.38 (63.05; 131.90)	0.117

Median and interquartile range (IQR); eGFR—estimated glomerular filtration ratio; M—male; F—female; FGF23—fibroblast growth factor-23; ACR—albumin-to-creatinine ratio.

**Table 3:** Spearman correlation between parameters, biochemical factors, and α-Klotho.

	r	*p*
Age at diagnosis (years)	−0.126	0.313
Age at study (years)	−0.366	0.002
Follow up after treatment (years)	−0.173	0.164
Methotrexate (cumulative dose (mg/m^2^))	−0.056	0.725
Prednisone (cumulative dose (mg/m^2^))	0.197	0.195
Dexamethasone (cumulative dose (mg/m^2^))	−0.050	0.753
Cyclophosphamide (cumulative dose (mg/m^2^))	−0.115	0.445
Cisplatin (cumulative dose (mg/m^2^))	−0.463	0.296
Serum creatinine	−0.321	0.010
Urine Creatinine	−0.182	0.154
ACR	0.155	0.226
eGFR	0.074	0.569

**Table 4:** Spearman correlation between parameters, biochemical factors, and FGF23.

	r	*p*
Age at diagnosis (years)	−0.160	0.199
Age at study (years)	0.158	0.206
Follow up after treatment (years)	0.278	0.024
Methotrexate (cumulative dose (mg/m^2^))	−0.458	0.002
Prednisone (cumulative dose (mg/m^2^))	−0.206	0.174
Dexamethasone (cumulative dose (mg/m^2^))	−0.124	0.435
Cyclophosphamide (cumulative dose (mg/m^2^))	0.015	0.920
Cisplatin (cumulative dose (mg/m^2^))	0.000	1
Serum creatinine	0.205	0.108
Urine Creatinine	0.412	0.001
ACR	−0.202	0.112
eGFR	−0.148	0.255

## Data Availability

The data that support the findings of this study are available from the corresponding author upon reasonable request. Some data may not be made available because of privacy or ethical restrictions.
